# Reconfigurable multi-scale colloidal assembly on excluded volume patterns

**DOI:** 10.1038/srep13612

**Published:** 2015-09-02

**Authors:** Tara D. Edwards, Yuguang Yang, W. Neil Everett, Michael A. Bevan

**Affiliations:** 1 Chemical & Biomolecular Engineering, Johns Hopkins University, Baltimore, MD 21218; 2 Exoteric Instruments, 604 Basie Bend, Cedar Park, TX 78613.

## Abstract

The ability to create multi-scale, periodic colloidal assemblies with unique properties is important to emerging applications. Dynamically manipulating colloidal structures *via* tunable *kT*-scale attraction can provide the opportunity to create particle-based nano- and microstructured materials that are reconfigurable. Here, we report a novel tactic to obtain reconfigurable, multi-scale, periodic colloidal assemblies by combining thermoresponsive depletant particles and patterned topographical features that, together, reversibly mediate local *kT*-scale depletion interactions. This method is demonstrated in optical microscopy experiments to produce colloidal microstructures that reconfigure between well-defined ordered structures and disordered fluid states as a function of temperature and pattern feature depth. These results are well described by Monte Carlo simulations using theoretical depletion potentials that include patterned excluded volume. Ultimately, the approach reported here can be extended to control the size, shape, orientation, and microstructure of colloidal assemblies on multiple lengths scales and on arbitrary pre-defined pattern templates.

Ordered multi-scale colloidal assemblies can generate periodic and resonant structures comparable to or smaller than wavelengths of electromagnetic radiation to create metamaterials^1^,^2^. Metamaterials have emergent aggregate material properties that affect their interaction with elecromagnetic radiation (*e.g*., absorbance, reflection, transmission) that are observed in nature (*e.g*., butterflies, beetles^3^,^4^) and could be created in synthetic materials for emerging applictions (*e.g*., sensing, solar cells, imaging, cloaking, antennas^5^,^6^,^7^). Colloidal assembly provides a possible method to fabricate large-area hierarchical structures required for metamaterials, including ensemble particle responses (that differ from single particles and bulk materials^8^) and patterning of resonant structures (*e.g.*, split rings)^1^,^9^. In addition to using colloidal assembly for metamaterial fabrication, reversible assembly schemes can enable switching multi-scale structures between two or more configurations as a basis to develop reconfigurable metamaterials for use in devices.

It is important to consider a number of factors to design colloidal interactions and assembly schemes suitable for reconfigurable multi-scale metamaterials. While colloids can easily be assembled into amorphous, irreversible, static, arrested structures by strong attractive interactions^10^ and can be coaxed into close-packed crystalline configurations by carefully tuning attractive interactions on the order of the thermal energy (*kT*)^11^,^12^, generating multi-scale structures is much more challenging. For example, it is possible to assemble space-filling, non-close packed configurations using templated sedimentation^13^, magnetic^14^,^15^ and electrostatic^16^,^17^ attraction, capillary forces^18^, laser tweezers^19^, chemical synthesis^20^, site-specific bonding^21^,^22^,^23^, spin coating^24^, and tri-block colloids^25^. Generating colloidal microstructures *via* tunable *kT*-scale attraction provides the opportunity for material/device reconfiguration, which has been achieved *via* external stimuli, including opto-electrical effects^26^,^27^, high frequency AC electric fields^28^,^29^,^30^,^31^, and thermosensitive depletion interactions^11^,^12^,^32^,^33^. For the specific case of depletion interactions, it has also been possible to develop local and directional attraction by designing local surface features^34^, templates for crystallization^35^,^36^, surface roughness^37^,^38^,^39^, and lock-and-key colloids (*i.e.*, local curvature)^40^.

In this work, we demonstrate the reversible assembly/disassembly of micron scale colloidal chains at the edges of ciruclar features patterned into an array, which represents a basic periodic arrangement of resonant structures that could be developed into a large-area reconfigrable metamaterial. In particular, we report a tactic to generate reconfigurbale multi-scale colloidal assemblies by combining thermosensitive depletion interactions and patterned surface topographical features that, together, reversibly mediate local *kT*-scale depletion interactions (Fig. 1a,b and Supplementary Fig. S1). In brief, depletion interactions, which rely on the exclusion of solute particles between surfaces, are manipulated by: (1) thermoresponsive poly-N-isoproplyacrylamide (PNIPAM) hydrogel depletant particles that alter depletion attraction *via* their temperature-dependent size, and (2) systematically varying physical surface patterns that lead to local excluded volume effects that mediate depletion attraction between colloids and pattern features. Using a model recently developed by us^41^ (including previous high-resolution particle-wall and particle-particle potentials^11^,^12^), we measure, analyze, simulate, and design depletant and pattern dimensions to understand the net interactions that control colloidal assembly.

While thermoresponsive depletion has been used to reversibly assemble close-packed colloidal crystals^12^,^32^,^33^, and surface patterns have been used to create local depletion attraction^34^ (including templates for close-packed colloidal crystals^11^,^35^,^36^), we are unaware of a precedent to exploit a synergistic combination of such methods to control multi-scale colloidal assembly. By investigating local depletion mediated interactions and assembly on model surface topographies, it is possible to understand the fundamental mechanisms underlying the net depletion interaction; it depends on both changing the depletant osmotic pressure and the excluded volume determined by the depletant size in combination with local topographical feature dimensions. Our results demonstrate how size tunable depletants produce comparable changes in solution osmotic pressure and local excluded volume to generate reconfigurable multi-scale colloidal assemblies on topographically patterned substrates. Ultimately, our findings include the development of accurate quantitative models of locally tunable depletion interactions that could enable the rational design, control, and optimization of particle based reconfigurable materials and devices.

## Results and Discussion

### Depletion interactions on patterned surfaces

**Figure 1:**
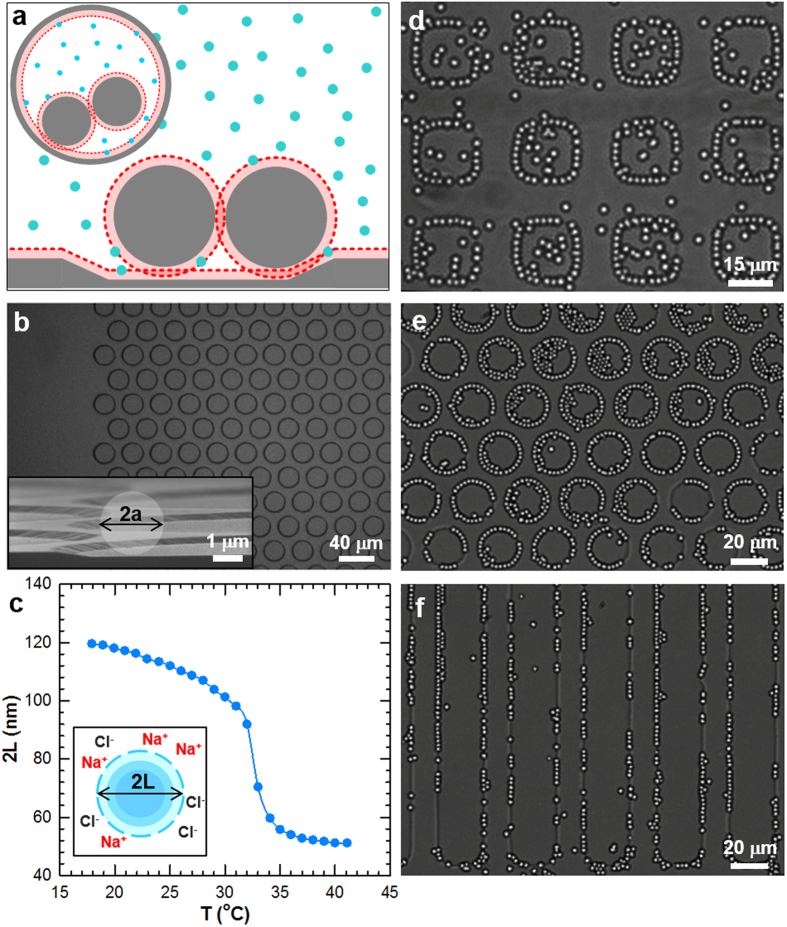
Reversible multi-scale assembly of charged ~2 μm diameter SiO_2_ colloids above topographical surface patterns resulting from thermoresponsive hydrogel nanoparticle-mediated depletion interactions. (**a**) Schematic of charged SiO_2_ colloids (gray circles) experiencing depletion attraction with each other and a topographical feature due to the exclusion of the smaller spherical depletants (cyan circles). When the surface-to-surface separation between particles or particles and the underlying feature is less than the depletant diameter, 2*L*, (light red areas) the depletants are excluded from the overlapping regions (darker red areas). Inset is a top-down view of SiO_2_ colloids experiencing depletion interactions with a circular patterned feature. (**b**) Microscopy image of etched circles arranged on a hexagonal lattice. Inset is an SEM image of a cross-section showing the resulting sidewall geometry with an overlaid representation of a silica colloid. (**c**) Hydrodynamic diameter of PNIPAM depletant nanoparticles in 1 mM NaCl as a function of temperature. (**d**–**f**) Experimental images extracted from video microscopy measurements of equilibrated multi-scale self-assembled SiO_2_ particles at 25 °C (PNIPAM *2L *≅ 113 nm) localized to the edges of ~285 nm deep (**d**) 20 μm × 20 μm squares, (**e**) 20 μm circles on a hexagonal lattice, and (**f**) 20 μm wide channels.

PNIPAM hydrogel nanoparticles were synthesized (see Supplementary Methods) for use as depletant particles. In aqueous media, their diameter, 2*L*, varies as a function of temperature, *T*. PNIPAM particles are swollen with water at low temperatures but become continuously desolvated as their lower critical solution temperature is approached. Dynamic light scattering measurements were used to measure the hydrodynamic diameter of the PNIPAM particles vs. temperature (Fig. 1c)^12^,^42^. The continuous change in PNIPAM size vs. temperature suggests a continuous “knob” for tuning depletion attraction and the associated self-assembly (rather than a “switch” for a first-order transition); we revisit whether a continuous transition in the assembly process is possible at the end of our discussion.

Video microscopy was used to directly measure quasi-two dimensional (2D) configurations of nominal 2*a *= 2.34 μm diameter SiO_2_ colloids with PNIPAM hydrogel depletant particles in 1 mM NaCl sedimented onto periodic arrays of physically patterned wells of different shapes and sizes. Gravity concentrates the SiO_2_ colloids within 285 nm deep patterned wells while 112.7 nm (*T *= 25 °C) diameter PNIPAM depletants caused the SiO_2_ particles to outline the inner well side walls. Figure 1d–f displays different types of periodic multi-scale colloidal structures obtained by combining depletion interactions with locally patterned excluded volume.

In all cases, assembly initiates along feature edges due to a local increase in excluded volume (*via* the sloped 13° walls, Supplementary Fig. S2b), and hence increased depletion attraction that localizes particles at feature edges^34^. Because particles also experience depletion attraction with each other, the localized particles first tend to form chains at pattern edges, and then layer into 2D crystalline states as more particles are added. Samples at lower colloid area concentrations form incomplete rings of partial chains *via* lateral particle-particle attraction (Fig. 1e), whereas higher concentrations produce multilayers within the wells (also in Fig. 1e). The average concentration inside and outside features is expected to have a Boltzmann dependent partitioning based on the free energy difference determined by gravity and multi-particle packing effects^11^,^43^. Finally, crystals unattached to the pattern edge were never observed for any combination of pattern depth, depletant size, or particle concentration described in the following study.

### Colloidal assembly vs. pattern feature depth

**Figure 2:**
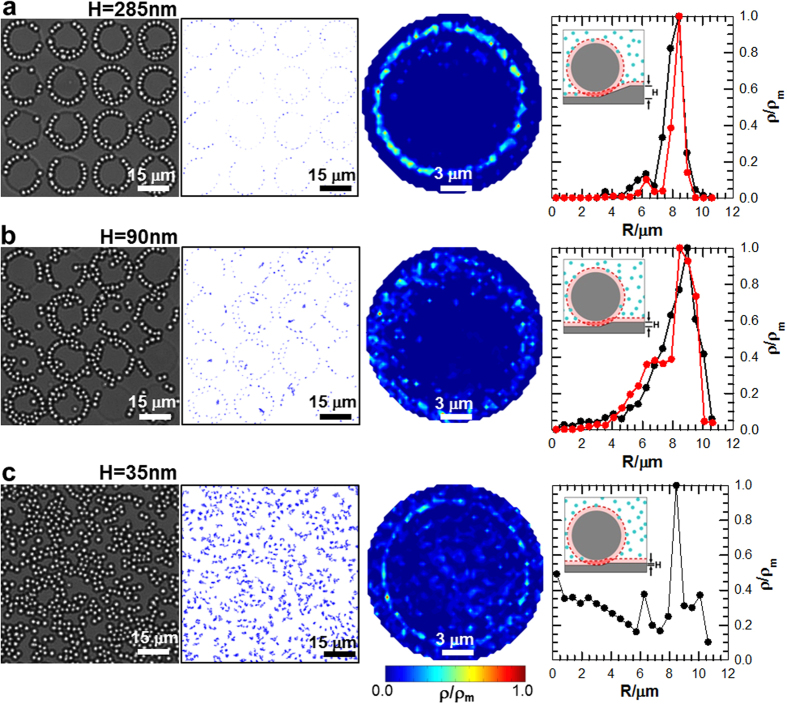
Structural, spatial, and statistical information obtained from video microscopy of equilibrated colloidal assembly as a function of topographical surface pattern depth. Results shown for patterned 12 μm circles arranged on a square lattice with (**a**) *H *= 285 nm, (**b**) *H *= 90 nm, (**c**) *H *= 35 nm deep at ~25 °C in the presence of ~113 nm diameter PNIPAM hydrogel nanoparticles. (left-to-right) Experimental images extracted from video microscopy, dynamic traces of 2D particle trajectories over patterns from the first 1,000 experimental frames (~36 s), contour plots of 2D density profiles with experimental particle center coordinates averaged over all features, and 1D radial density profiles from experiments (red) and MC simulations (black) (except for *H *= 35 nm experiment where pattern edges could not be resolved).

Colloidal assembly as a function of well depth was investigated by using the same system described in Fig. 1d–f with features etched to various depths. The far left column in Fig. 2 shows images of colloidal configurations extracted from video microscopy measurements (Supplementary Videos S1–S3) for three different well depths: *H *= 285 nm, 90 nm, and 35 nm. Consistent with the results in Fig. 1d–f, Fig. 2a,b shows colloidal SiO_2_ outlining the inner periphery of features within 285 nm and 90 nm deep wells (again, with multilayers forming in some cases *via* particle-particle depletion attraction). In Fig. 2c, where *H *= 35 nm, the additional excluded volume at feature edges becomes negligible, and particles formed an equilibrium fluid microstructure across the entire substrate surface, demonstrating that particle-particle depletion attraction was insufficient to induce quasi-2D crystallization in the absence of significant surface topographical features.

The second column of Fig. 2 shows 2D particle trajectories for the first ~36 s extracted from video microscopy measurements for the three well depths. These plots trace the positions sampled by each particle within each well feature as a function of time to convey the degree of particle motion vs. feature depth (for readers without access to Supplementary Videos S1–S3). The data at *H *= 285 nm shows that particles are arranged near the inside perimeter of the features. At *H *= 90 nm, occasional particle excursions away from the sidewalls can be observed, but otherwise, the plots indicate that the particles prefer to be situated proximal to other particles and along the interior well edges. At *H *= 35 nm, trajectories in Fig. 2c show particles diffusing over the patterned surface with only minor effects of particle-particle attraction and gravity.

To model the combined colloid, depletion, and patterned surface interactions as a function of *H*, Monte Carlo (MC) simulations were performed at each of the three well depths using a novel method reported by us elsewhere^41^. Details of the model are provided in this previous paper, but we provide some key information here for convenience. The depletion potential, *U*_D_, vs. seperation, *r*, was modeled using a modified form of the usual Asakura-Oosawa (AO) depletion potential as^12^,^42^,^44^,


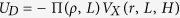


where the depletant osmotic pressure, Π, and excluded volume, *V*_X_, both depend on the thermoresponsive depletant size, *L*, and *ρ* is the depletant number density. Direct measurements have shown this potential accurately captures particle-wall and particle-particle depletion interactions (for a variety of material systems)^12^ using the Carnahan-Starling hard sphere equation of state^45^ for Π (accurate equations of state are critical to quantitative depletion potentials^42^) and the usual hard sphere excluded volume terms^46^. We use the particle-particle and particle-wall potentials from this previous work without modification, since they were shown to accurately capture particle-wall potentials at high-spatial resolution and infinite dilution as well as quasi-2D phase behavior without any adjustable parameters^11^,^12^. In this work, *V*_X_ is computed for particles and underlying surfaces using the same hard sphere expressions; however, it is computed numerically to consider local pattern features (see Supplementary Theory)^41^. The origin and value of all independently measured parameters used in interaction potentials (see Supplementary Theory Eq. (S1)-(S8)) are reported in Table 1.

Because *V*_X_ depends on the surface-to-surface separation, *r*, between colloidal particles and the substrate, for particles interacting with a pattern feature’s side wall, it also depends on the pattern feature’s height, *H*. The potential for *U*_D_ indicates that at a given temperature (*i.e*., fixed depletant size, *L*), the value of Π is fixed and *V*_X_ increases with increasing *H* for *r *< 2*aL*; this increases depletion attraction between particles and patterned features. This is verified by the agreement of our experimental and simulation results in Fig. 2 that show depletion attraction between colloidal particles and pattern edges decreasing with decreasing feature depth.

To capture time-averaged particle microstructures, quasi-2D particle density landscapes, *ρ*(*x*, *y*), were constructed relative to each circular pattern center by averaging particle center coordinates over 16 pattern features in both experiments and MC simulations for each well depth^43^. In this work, it is not necessary to consider other distribution functions since the particle-wall and particle-particle potentials are already known from previous work^11^,^12^. The third column of Fig. 2 gives contour plots of *ρ*(*x*, *y*) from the experiments normalized by the maximum density, *ρ*_m_, for the three well depths investigated. A ring of density at the radial distance corresponding to particle centers about one radius away from the pattern edge clearly captures particle attraction to this feature.

In addition to the radial density dependence showing particles at the pattern feature edge, there is a slight angular dependence (as indicated by the peaks in the experimental landscapes) within the radial ring closest to the edge. This suggests some structural correlation or alignment between adjacent patterns. This effect is very weak and does not create any issues when matching simulations and experiments using the reported potential for *U*_D_, but could arise from: (1) insufficient averaging to generate perfect 2D distribution functions, (2) a component of gravity parallel to the surface due to mis-leveling, (2) weak convection causing shear alignment during assembly, (3) some small scale surface texture resulting from the wet etching patterning method producing local excluded volume^37^,^38^,^39^. However, with regard to the latter effect, the Supplementary Videos also clearly show single particles coexisting with particle chains that are freely mobile along the pattern edges demonstrating that roughness is insufficient to localize particles at specific angular positions. Aside from the small angular density fluctuations, the experimental and MC simulated 2D density landscapes are indistinguishable within the experimental resolution limits and materials nonuniformities (*e.g.*, particle polydispersity, pattern heterogeneity).

Density landscapes for the two deeper well depths, *H *= 285 nm and 90 nm, in Fig. 2a,b show particles concentrated around the interior periphery of pattern features and that there are very few, if any, excursions of the particles away from the sidewalls or each other. However, whereas particle-surface interactions keep the ring of particles in the first layer around the inside pattern edges immobile, the second layer of particles appear to intermittently break free and reform long lifetime bonds for *H *= 90 nm but not for *H *= 285 nm. Because the particle-particle depletion attraction does not obviously depend on *H*, it appears that a weakened particle-edge attraction allows more thermal motion in the first layer that occasionally causes a particle in the second layer to break free. This effect is accurately captured in the MC simulations without any modifications to the particle-particle depletion attraction, which supports an explanation of a multi-particle packing effect in conjunction with thermal motion in the first layer.

The density landscape for the shallowest well depth (*H *= 35 nm), where there is minimal depletion attraction, shows that gravity slightly concentrates the particles to the inside of the circular wells. The concentration inside the feature is determined by a Boltzmann distribution dependent on the free energy difference of residing at the lower elevation inside the well compared to outside the pattern feature^11^,^43^. Otherwise, the particles are randomly distributed over the pattern surface as an inhomogeneous fluid phase.

The 2D density profiles in Fig. 2 can be plotted as one-dimensional (1D) density landscapes (far right column) by converting to polar coordinates and averaging over the angular coordinate. Results are shown for both the microscopy experiments and Monte Carlo simulations, except for the *H *= 35 nm experiment where the pattern edges were difficult to resolve under the randomly distributed particles. These results most clearly quantify how the particle density varies relative to the patterned feature edge vs. feature depth. Good correspondence is observed between the experimental and simulations results. The presence of multiple peaks in the 1D density profiles of Fig. 2a–c captures particle packing effects. The 1D density profiles of Fig. 2a and b for *H *= 285 nm and 90 nm have a maximum peak occurring at the inside edge of the pattern feature *R*, and a secondary peak at ~1.5*a* toward the feature center. This verifies that particles are more attracted to the inside perimeter of the pattern features and to each other when the well depth is between *H *= 285 nm and 90 nm^41^. The 1D density profiles of Fig. 2c have multiple peaks corresponding to particle clusters distributed inhomogeneously over the surface. Thus, at *H *= 35 nm, there is not enough attraction for the particles to display a significant energetic preference for feature edges.

At this point, we note that although the sloped side walls of the pattern features exhibit some roughness (*e.g.*, Fig. 1b inset), which could potentially increase the local excluded volume and depletion attraction^37^,^38^,^39^, modeling the sloping wall edges as flat planes produces excellent agreement between simulations and experiments^41^. This finding indicates the effect of the roughness is negligible and makes sense based on the relative size of the surface asperities compared to the colloidal particles and depletant particles. In particular, the roughness is sufficiently small that it does not change how depletants are excluded between the colloidal particles and the pattern feature compared to smooth surfaces.

### Colloidal assembly vs. depletant size

**Figure 3:**
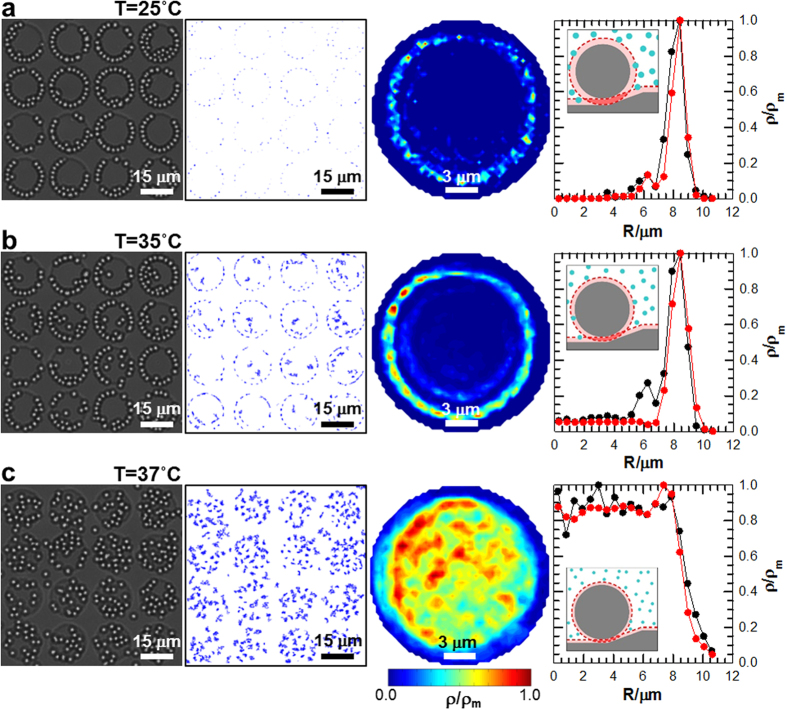
Structural, spatial, and statistical information obtained from video microscopy of equilibrated colloidal assembly as a function of temperature (*i.e.*, depletant size). Results shown for patterned 12 μm diameter circles arranged on a square lattice with *H *= 285 nm deep in the presence of PNIPAM hydrogel nanoparticle depletants at (**a**) 25 °C (2*L *≅ 113 nm), (**b**) 35 °C (2*L *≅ 107 nm), and (**c**) 37 °C (2*L *≅ 53 nm). (left-to-right) Experimental images extracted from video microscopy, dynamic traces of 2D particle trajectories over patterns from the first 1,000 experimental frames (~36 s), contour plots of 2D density profiles with experimental particle center coordinates averaged over all features, and 1D radial density profiles from experiments (red) and MC simulations (black).

Based on PNIPAM’s *T*-dependent size (Fig. 1c)^11^,^12^, we investigated assembly at feature edges vs. *T* for arrays of 285 nm deep wells. The depletion attraction, *U*_D_, decreases with decreasing depletant size since both Π and *V*_X_ decrease with decreasing depletant size. Depletant size decreases with increasing *T* in the present work so that *U*_D_, Π, and *V*_X_ also all decrease with increasing *T*. This is tested for a fixed well depth of *H *= 285 nm in Fig. 3 and Supplementary Videos S4–S6. Figure 3 shows in a similar format to Fig. 2 how particle ordering varies at three different *T*. By switching the temperature between 25 °C and 37 °C, it was possible to control the degree of colloidal ordering within the pattern features and reconfigure colloidal assemblies between disordered fluid and multi-scale patterned states. The change in particle assembly with changing *T* in Fig. 3 occurred in ~10 min. A total of ~30 min was allowed for equilibration before analyzing particle configurations for Fig. 3. This process was completely reversible for all pattern feature sizes, depths, and geometries in this work.

In Fig. 3a, snapshots from video microscopy clearly show that particles outline the inside edges of the patterned circular well features at 25 °C, as was previously shown in Figs 1d–f and 2a,b. Again, colloidal concentrations greater than that needed to entirely outline the inside circumference of the pattern feature resulted in the start of a second ring of particles within the first. Likewise, no freestanding crystals were observed in the pattern interior. Colloidal 2D trajectories display the degree of particle motion away from the inner pattern feature edge as a function of time. The 2D trajectory plot of the second column in Fig. 3a shows that particles at 25 °C deviate minimally from the inside periphery of the wells. At this temperature, PNIPAM depletant particles are 113 nm in diameter (Fig. 1c), which gives rise to a significant depletion attraction at the well periphery and colloidal chain assembly.

Similar to the well depth results in Fig. 2a, particle-particle depletion attraction at this temperature is also significant relative to *kT* so that the 2D colloidal trajectories show particles comprising the second ring are also immobile. In Fig. 3b, at *T *= 35 °C (2*L *≅ 107 nm), the depletion attraction is reduced (*i.e.*, maximum particle-wall attraction decreases from 19*kT* at 25 °C to 10*kT* at 35 °C and particle-particle attraction decreases from 8*kT* to 3*kT*, respectively). Video microscopy images of equilibrium particle positions and 2D particle trajectories on the patterned substrate demonstrate sufficient depletion attraction for assembly, although a small increase in particle diffusion provides some evidence for a temperature-dependent reduction in the attraction. The 2D particle trajectories also show particles within the second layer in pattern features occasionally attaching and detaching due to reduced particle-particle depletion attraction. Finally, at 37 °C when depletant particles deswell to 2*L *≅ 53 nm (Fig. 3c), depletion attraction is negligible, and particles diffuse freely except for the small gravitational energy difference inside and outside features^43^.

To quantify the spatially varying particle distributions in these experiments, quasi-2D density landscapes, *ρ*(*x*, *y*)/*ρ*_m_, were constructed from particle centers from video microscopy experiments averaged over ~30 min at each *T*. As in Fig. 2, assuming all pattern features to be identical within microfabrication limits, the density landscapes were averaged over all features. The resulting time-averaged 2D density landscapes appear as contour plots in the third column of Fig. 3. The density landscapes clearly show particles: assembling along feature edges at 25 °C, assembling but with increased local thermal motion at 35 °C, and disassembling with uniform diffusion within wells at 37 °C. The density landscapes capture the slight substrate tilt, evidenced by the increased sampling of the upper left quadrant of each feature. This illustrates how it is possible to bias the location of colloidal chain assembly within each feature with a lateral gravitational potential or another external field (which may be related to the weak angular correlations in Fig. 2).

To quantify how temperature-dependent depletion affects colloidal assembly within features, MC simulations were performed^41^, using the parameters reported in Table 1. One-dimensional density profiles constructed from time-averaged particle coordinates are plotted in Fig. 3a–c to show radial particle density vs. temperature. For *T* = 25 °C and 35 °C in Fig. 3a and b, respectively, the 1 D density shows a maximum peak at the feature sidewall and a secondary peak at ~1.5*a*, resulting from colloidal assembly in the pattern interior. Although the secondary interior peak is absent from the experimental profile in Fig. 3b, we attribute this discrepancy to inadequate statistical sampling in the experiment (*i.e.*, accurately quantifying this small peak depends strongly on observing a small number of particles bound to the interior of colloidal chains attached to the pattern edge). In contrast, the peak is clearly present in the experiments at *T *= 25 °C in Fig. 3a for stronger particle-particle attraction.

The 1D density profile at 37 °C in Fig. 3c shows that the particle density is evenly distributed within wells as the result of vanishing depletion attraction. The 1D density profiles in Figs 2 and 3 from experiments and MC simulations show excellent agreement within the spatial resolution and sampling limits in the optical microscopy measurements^47^ as well as the particle and microfabricated pattern uniformity^43^,^48^,^49^,^50^,^51^.The results in Figs 2 and 3 generally validate the interpretive/predictive capability of the interaction potential for *U*_D_ and our numerical routine for computing excluded volume^41^.

### Colloidal assembly vs. local depletion attraction

**Figure 4:**
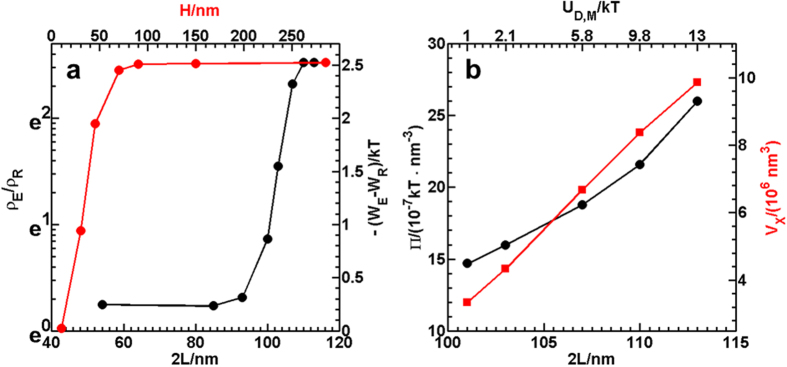
Density, free energy, and potential energy (with osmotic pressure/excluded volume contributions) for particles at pattern feature edge vs. depletant size and feature depth. (**a**) Density at pattern edge, *ρ*_E_, relative to density on pattern interior as reference state, *ρ*_R_, vs. depletant size, 2*L*, for *H *= 285 nm and vs. pattern depth, *H*, for 2*L *= 113 nm. Ratio of densities plotted on the right-hand-side on an exponential scale to show Boltzmann relationship, *ρ*_E_/*ρ*_R _= exp[−(*W*_E_ − *W*_R_)/*kT*], to the left-hand-side showing free energy difference between particles at the pattern edge, *W*_E_, compared to the pattern interior, *W*_R_ (*i.e.*, reference state). (**b**) Osmotic pressure, *Π*, and excluded volume, *V*_X_, vs. depletant size, 2*L*, and vs. depletion attraction minimum, *U*_D,M_, based on particle-wall depletion attraction, which is related by a factor to depletion attraction at the pattern edge (*i.e.*, pattern edge *V*_X_ can be considered as some combination of 2 walls) for different *H* and wall angles. Note that the scale for *U*_D,M_ has a non-linear relationship to the 2*L* scale and is plotted as text to simply show values corresponding to *U*_D,M_.

We now use the MC simulations matched to experiments to further explore in Fig. 4 how the density of particles attached to the pattern feature edge, *ρ*_E_, varies as a function of depletant size and well depth. In particular, we invstigate more values than were probed in the experiments to get a better sense of how the assembly transitions from disordered fluids to chains lining pattern edges. In Fig. 4, we are also able to relate density changes in assembled particles at the pattern edge to the free energy and potential energy of colloid attachment to the well edge, as well as changes in osmotic pressure and excluded volume.

Figure 4a plots *ρ*_E_/*ρ*_R_ vs. depletant size, 2*L*, for *H *= 285 and vs. pattern depth, *H*, for 2*L *= 113 nm, where *ρ*_R_ is the reference density in the pattern interior. Values of *ρ*_E_ and *ρ*_R_ are taken directly from MC simulated 1D densitry profiles like those reported in column 4 of Figs 2 and 3. The quantity *ρ*_E_/*ρ*_R_ is plotted on an exponential scale so it can be related to a second linear axis for the free energy of attachment of particles to the pattern edge *via* a Boltzmann relationship, *ρ*_E_/*ρ*_R _= exp[−(*W*_E_ − *W*_R_)/*kT*]. In this relationship, *W*_E_ − *W*_R_ is the difference in the free energy of particles at the pattern edge, *W*_E_, compared to the free energy of particles in the pattern interior, *W*_R_ (*i.e.*, the reference state in this case).

The quantities *ρ*_E_/*ρ*_R_ and −(*W*_E_ − *W*_R_)/*kT* in Fig. 4a show a sharp transition of particles not being associated with the pattern edge or each other (no obvious attraction) to being highly localized at the edge in chains (attraction to the edge and other particles) for changes in both 2*L* and *H*. As such, the reconfiguration mechanism associated with colloidal chain assembly at the topographical pattern edges has a first-order “type” transition both with changing depletant size and changing pattern well depth. Although a truly one-dimensional fluid-solid transition is not expected to display first-order behavior, quasi-1D freezing experiments and simulations routinely show abrupt phase changes indistinguishable from first-order transitions^52^. Because the particle-topographical pattern interaction in this work is clearly 3D in nature, it does not make sense to consider our observation of disordered fluid states condensing into near equilibrium colloidal chains as a 1D or even quasi-1D phenomenon. As such, the observation of a first-order “type” of transition between fluids and patterned chains is not at odds with expectations for 1D phase transitions (that are not obviously applicable to this work).

We can examine *U*_D_ more closely to better understand the mechanism associated with depletant size producing a relatively sharp transition in the reconfiguration of particles between inhomogeneous fluids and patterned chains. Figure 4b plots the PNIPAM solution Π vs. 2*L* using the Carnahan-Starling hard sphere equation of state^45^. Figure 4b also shows the particle-wall *V*_X_ vs. 2*L* using the standard particle-wall equation^46^ (also see Eq. (S5)) for separations values of *z*_m_ in Table 1, which is the particles’ most probable height above the substrate due to a balance of gravity and electrostatic repulsion (see Supplementary Theory and Eq. (S6)). For the feature geometry used in this work (see Fig. 1b), the feature *V*_X_ is always approximately some factor times the particle-wall value; for example, it is ~1*V*_X_ as the angle goes to zero and closer to ~2*V*_X_ (in some cases) as the angle goes to 90°. As such, the percent change in the particle-wall *V*_X_ will also be the percent change in the particle-feature *V*_X_, even if the absolute values differ. On the top axis, the values of the net potential minimum, *U*_D,M_, are plotted vs. 2*L*, which does not have a straightforward linear relationship, so they are simply plotted as text values for reference. In short, as the depletant sizes changes from 100 nm to 115 nm, the depletion attraction changes from 1*kT*–13*kT*. This is the result of Π increasing ~2× and *V*_X_ increasing ~3× over the same range of depletant size.

The results in Fig. 4, taken together, show that the potential indeed changes continuously vs. depletant size with comparable effects due to the changes in Π and *V*_X_, which also change continuously. Because 2*L*, Π, *V*_X_, and *U*_D,M_ all change continuously, ultimately the abrupt change from fluid states to patterned chains occurs with a critical level of attraction in what appears to be a first-order “type” transition. At least for the types of tunable depletants and patterned excluded volume investigated in this work, the colloidal assembly undergoes a switching behavior to enable a potential two-state metamaterial with on- and off- configurations. More continuous or multi-state transitions could eventually enable further tuning of multi-scale metamaterials.

In conclusion, we present a novel approach to reconfigurable colloidal assembly of multi-scale structures that combines thermoresponsive depletion attraction with topographically patterned surface features. Changing both the temperature and topography was shown to reconfigure colloidal microstructures between two well-defined states: multi-scale periodic structures and disordered fluid states. Spatial- and time-averaged density profiles characterized how the degree of colloidal ordering can be controlled as a function of patterned feature depth and temperature, which was quantitatively captured by theoretical depletion potentials. Our findinsg show the formation of patterned colloidal chains occurs *via* a first-order “type” transition depsite the continously tunable depletant size, osmotic pressure, excluded volume, and local depletation attraction. The ability to dynamically manipulate multi-scale colloidal assemblies provides a basis for the development of micro- and nano- scale periodic and resonant structured materials and devices. Ultimately, the approach reported here can be extended to control the size, shape, orientation, and microstructure of colloidal assemblies on multiple lengths scales and in arbitrary pre-defined pattern templates.

## Methods

### Surface patterning

Topographical features were wet etched into microscope slides (Gold Seal, VWR), using patterned photoresist (S1818, Microchem) as the etch mask. All etching was carried out under agitation at 25 °C in a mixture of oxide etchants (1:1 Timetch:Buffer-HF-Improved, Transene Company), with the resulting depth controlled by etch time. Feature depths were measured with a stylus profilometer (Dektak 6M) at ten positions across the array. The feature sidewalls had sloping edges due to the isotropic nature of the wet etch (Fig. 1b inset and Supplementary Fig. S2).

### Particles

Nominal 2.34 μm diameter SiO_2_ colloids (Bangs Laboratories) and NaCl were used as received. Uncharged PNIPAM hydrogel particles were synthesized using a standard literature procedure (see Supplementary Methods)^53^. Patterned microscope slides were sonicated in acetone, isopropanol, and then deionized (DI) water, followed by 1 h of immersion in piranha (3:1 H_2_SO_4_:30% H_2_O_2_,), a DI water rinse, sonication in 100 mM KOH for 30 min, a DI water rinse, and then dried with N_2_ prior to each experiment. Dispersions of 2.34 μm SiO_2_, PNIPAM, aqueous NaCl, and DI water were prepared to yield 1 mM NaCl with a 17.5% stock PNIPAM volume fraction and ~18% SiO_2_ interfacial area fraction.

### Video microscopy

All experiments were performed in ~100 μm thick, epoxy-sealed sample cells consisting of a microscope slide substrate and a glass coverslip. Temperature control was achieved using a heating stage with a Bionomic System BC-110 temperature controller (20/20 Technology, Inc.). Patterned substrates were coupled to the heating stage using a silicone-based heat sink compound (RadioShack). A 12-bit CCD camera (ORCA-ER, Hamamatsu) on an inverted optical microscope (Axio Observer A1, Zeiss) was operated in 4-binning mode with a 63× objective to yield 28 frame/s and 385 nm/pixel. Image analysis algorithms coded in Fortran were used to track lateral colloid motion in all experiments^11^,^43^,^51^,^54^.

### Monte Carlo simulations

VM experiments were modeled in 2D MC simulations, where colloids sampled landscapes determined by gravity and substrate topography-mediated depletion potentials, using a novel approach to compute local excluded volume effects, which is described in detail elsewhere^41^. MC simulations were performed using parameters provided in Table 1, and initial configurations were obtained from experimental images. Each MC simulation was performed for 2.5 × 10^6^ steps with particle positions stored every 250 steps after an initial equilibration of 10^6^ steps.

## Additional Information

**How to cite this article**: Edwards, T. D. *et al.* Reconfigurable multi-scale colloidal assembly on excluded volume patterns. *Sci. Rep.* **5**, 13612; doi: 10.1038/srep13612 (2015).

## Supplementary Material

Supplementary Information

Supplementary Video 1

Supplementary Video 2

Supplementary Video 3

Supplementary Video 4

Supplementary Video 5

Supplementary Video 6

## Figures and Tables

**Table 1:** Parameters for experimental analysis and MC simulations in Figs 2, 3, 4 at each etched circular pattern well depth, *H*, and each temperature, *T* (*i.e.*, depletant diameter, 2*L*).

**Variable**	**Value**
*a*^a^ [nm]	1100
*ρ*_p_^b^[g cm^−3^]	1.92
*ρ*_f_^c^ [g cm^−3^]	1.0
κ^−1^^d^ [nm]	9.5
ψ^e^ [mV]	–50
T^f^ [°C]	25, 25, 25, 25, 35, 37
2L^g^ [nm]	113, 113, 113, 113, 107, 53
2R^h^ [μm]	17
D^i^ [μm]	21
H^j^ [nm]	35, 90, 285, 285, 285, 285
N^k^	672, 332, 332, 332, 332, 324
ϕ_A_^l^	0.36, 0.18, 0.18, 0.18, 0.18, 0.18
z_m_^m^	61, 61, 61, 61, 66, 100
Π^n^ [kT 10^−6^ nm^−3^]	2.6, 2.6, 2.6, 2.6, 1.8, 0.54
*ρ*_m_^o^ [μm^−2^]	7.46e^−3^, 2.10e^−2^, 2.03e^−2^, 2.26e^−2^, 1.17e^−2^, 6.67e^−4^

^a^particle radius previously obtained from measured gravitational potential using Total Internal Reflection Microscopy^12^;

^b^SiO_2_ colloid density (for Stöber SiO_2_)^55^;

^c^water density;

^d^Debye screening length calculated (Eq. (S3)) and from conductivity;

^e^particle and wall electrostatic potential previously fit to potential energy profiles in previous work^48^,^56^;

^f^temperature from *in situ* thermocouple;

^g^depletant particle diameter from dynamic light scattering (Fig. 1c);

^h^patterned circle diameter and

^i^center-to-center pattern spacing from scanning electron microscopy;

^j^patterned well depth from ten averaged profilometry measurements;

^k^number of particles from image analysis;

^l^colloid area fraction from image analysis;

^m^most probable height calculated using Eqs. (S6)-(S7);

^n^osmotic pressure calculated using Carnahan-Starling equation of state^11^,^12^,^45^;

^o^maximum colloid density value from image analysis.
