# Supplementary material for: Reconfigurable multi-scale colloidal assembly on excluded volume patterns
Source: Sci Rep. 2015 Sep 2;5:13612. doi: 10.1038/srep13612 (PMC4557032; doi:10.1038/srep13612)
Supplement: Supplementary Information [file srep13612-s1.pdf]

Reconfigurable multi-scale colloidal assembly on excluded volume patterns

Tara D. Edwards[1], *Yuguang Yang*[1], *W. Neil Everett*[2], *Michael A. Bevan*[1]*

Supplementary Methods

Synthesis of poly(N-isopropylacrylamide) hydrogel nanoparticles

Poly(N-isopropylacrylamide) (PNIPAM) hydrogel nanoparticles were synthesized using a standard precipitation polymerization method[1]. A mixture of 2 g of >99% N-isopropylacrylamide (NIPAM, Sigma-Aldrich Company, St. Louis, MO) monomer, 25 mg of 99% N-N-methylenebisacrylamide (MBA, Sigma-Aldrich Company, St. Louis, MO), and 0.18 g of 98.5% sodium dodecyl sulfate (SDS, Sigma-Aldrich Company, St. Louis, MO) were weighed out and added to a clean 300 mL round bottom flask with 98 mL of deionized (DI) water. The flask was subsequently sealed and submerged in a water bath with a thin layer of silicone oil (Fisher Scientific, Pittsburgh, PA) floating on top to prevent evaporation during heating. The reaction solution inside of the flask was degassed with nitrogen using a long stainless steel needle to remove excess oxygen. The nitrogen was bubbled into the reaction solution at a rate of approximately one bubble per second. The reaction mixture was also stirred constantly at 500 rpm using an egg-shaped PTFE coated magnetic stir bar (Fisher Scientific, Pittsburgh, PA). The water bath was heated to a temperature of 70 °C. The temperature of the water bath was checked periodically using a thermocouple.

Meanwhile, a solution of 25 mg of 99% potassium persulfate (KPS, Sigma-Aldrich Company, St. Louis, MO) dissolved in 2 mL of DI water was prepared. After at least 30 min when the water bath reached 70 °C, the KPS solution was injected into the reactor using a 10 mL syringe and needle (BD, Franklin Lakes, NJ) to initialize the polymerization reaction. The reaction was carried out for 4 h with constant stirring of the reaction solution at 300 rpm and at a bath temperature of 70 °C. After 4 h, the reactor was allowed to cool to room temperature, at which point the round bottom flask was stoppered and removed from the reactor set-up. The round bottom flask was stored at 2-8 °C for 48 h to allow the resulting PNIPAM hydrogel nanoparticles to swell.

The PNIPAM hydrogel nanoparticles were purified *via* dialysis against deionized (DI) water for 7 days at room temperature with the DI water changed once per day. The regenerated cellulose dialysis tubing had a molecular-weight cutoff of 12,000-14,000 (Fisher Scientific, Pittsburgh, PA). The nanoparticles were then concentrated by ultracentrifugation at 288,000 gravity and 41,000 rpm for 10 days at 25 °C (L7-65 Beckman Ultracentrifuge with SW 41 Ti titanium head swinging-bucket rotor, Beckman Coulter, Fullerton, CA). The supernatant was then poured off the clear gel network of PNIPAM nanoparticles that formed from ultracentrifugation in the bottom of the centrifuge tubes. Two mL of DI water was added to each centrifuge tube containing the gels and they were left to swell and redisperse at 2-8 °C for 3 days. To break up the gel network that formed during ultracentrifugation and to improve redispersion, the hydrogel nanoparticles were agitated *via* sonication in an ice water bath and/or vortexed.

The size of the nanoparticles was measured using dynamic light scattering (DLS) *via* a ZEN3600 ZetaSizer Nano (Malvern Instruments, Worcestershire, U.K.). The hydrodynamic

[1] Chemical & Biomolecular Engineering, Johns Hopkins University, Baltimore, MD 21218
[2] Exoteric Instruments, 604 Basie Bend, Cedar Park, TX 78613

* To whom correspondence should be addressed. email: mabevan@jhu.edu

diameter of the particles in 0.02 μm filtered DI water was found as a function of temperature (see Fig. 1c and Table 1). At 20 °C, the particles had a diameter of approximately $2L$ = 118 nm, and at 40°C, the particles had a diameter of approximately $2L$ = 51 nm.

Supplementary Theory

Net Interaction Potential

The net potential energy for colloidal particles interacting with each other, an underlying surface, and gravity (see Fig. 1a) can be modeled as the superposition of independent potentials. For a charged colloidal particle i with radius, a, in the presence of non-adsorbing depletant particles, the net interaction potential is given by,

$$U_i(\mathbf{r}_i) = U_D^{pw}(\mathbf{r}_i) + U_E^{pw}(\mathbf{r}_i) + U_G^{pf}(z_i) + \sum_{j \neq i} \left[U_D^{pp}(r_{ij}) + U_E^{pp}(r_{ij}) \right] \tag{S1}$$

where $\mathbf{r}_i=(x_i, y_i, z_i)$ is position vector of particle i, z_i is the particle center-to-surface elevation relative to the underlying surface, and r_{ij} is center-to-center separation between particles i and j. Subscripts refer to: (E) electrostatic, (G) gravitational, and (D) depletion, and superscripts refer to: (pp) particle-particle, (pw) particle-wall, and (pf) particle-field. The range of electrostatic repulsion in this work is sufficient so that van der Waals interactions van be neglected.

Gravitational Potential

The gravitational potential energy of each particle depends on its elevation above the reference surface multiplied by its buoyant weight, G, given by,

$$U_G^{pf}(z) = Gz = (4/3)\pi a^3 (\rho_p - \rho_f) gz \tag{S2}$$

where g is acceleration due to gravity, and ρ_p and ρ_f are the particle and fluid densities.

Electrostatic Interaction Potentials

The colloidal particles are electrostatically stabilized against aggregation and deposition due to van der Waals attraction. The interaction between electrostatic double layers on adjacent particle and planar wall surfaces are given by[2],

$$\begin{gathered} U_E^{pp}(r) = B \exp\left[-\kappa(r-2a)\right], \; U_E^{pw}(z) = 2B \exp\left[-\kappa(z-a)\right] \\ B = 32\pi\varepsilon a \left(\frac{kT}{e}\right)^2 \tanh\left(\frac{e\psi_1}{4kT}\right) \tanh\left(\frac{e\psi_2}{4kT}\right), \; \kappa = \left(2e^2 N_A C / \varepsilon kT\right)^{1/2} \end{gathered} \tag{S3}$$

where κ is the inverse Debye length, ε is the solvent dielectric constant, k is Boltzmann's constant, T is absolute temperature, C is the 1:1 monovalent electrolyte molarity, N_A is Avogadro's number, e is the elemental charge, and ψ_1 and ψ_2 are the surface potentials.

AO Depletion Potentials

The depletion attraction between particles and underlying substrate surface are modeled by a modified form of the usual AO depletion potential given by[3],

$$\begin{gathered} U_D^{pp}(r) = -\Pi(\rho, L) V_X^{pp}(r, L) \\ U_D^{pw}(\mathbf{r}) = -\Pi(\rho, L) V_X^{pw}(\mathbf{r}, L) \end{gathered} \tag{S4}$$

The excluded volume for particle-particle and particle-planar wall geometries, $V_X^{pp}(r, L)$ and $V_X^{pw}(\mathbf{r}, L)$, respectively (see Fig. 1), are given as[4-6],

$$V_X^{pp}(r,L)=\pi\left[(4/3)(a+L)^3\left(1-(3/4)r(a+L)^{-1}+(1/16)r^3(a+L)^{-3}\right)\right]$$
$$V_X^{pw}(z,L)=\pi\left[(4/3)L^3+4L^2a-4La(z-a)+a(z-a)^2-L(z-a)^2+(1/3)(z-a)^3\right] \quad \text{(S5)}$$

where L is the depletant radius. For particles in the vicinity of a topographical pattern feature, the excluded volume term in Eq. (S4) is computed numerically as described in one of our previous publications[7]. The numerical method has been validated in that it produces the correct excluded volume term for particle-particle and particle-wall geometries as in Eq. (S5).

Quasi-Two Dimensional Models

In this paper, we perform quasi-2D measurements, simulations, and analyses of particles on patterned surfaces. The particles are assigned to a most probable elevation, z_m, above the substrate, which is the location of the net potential energy profile minimum normal to the substrate where the sum of the forces on the particle equals zero. This value can be determined as the value of z where the gradient of the net potential energy (z-dependent potentials in Eq. (S1)) equals zero as given by,

$$\frac{\partial}{\partial z}\left[U_G^{pf}(z)+U_D^{pw}(z)+U_E^{pw}(z)\right]=0 \quad \text{(S6)}$$

This approach allows the position-dependent elevation of each particle, $z(\mathbf{r}_i)$, to be given as the sum of the local physical topography and the most probable height above the surface as,

$$z_i(x_i,y_i)=z_m+z(x_i,y_i) \quad \text{(S7)}$$

which gives the net quasi-two dimensional potential energy (including a quasi-2D gravitational potential energy landscape[8,9]) for each particle as,

$$U_i(\mathbf{r}_i)=Gz_M(\mathbf{r}_i)+U_D^{pw}(\mathbf{r}_i)+\sum_{j\neq i}\left[U_E^{pp}(r_{ij})+U_D^{pp}(r_{ij})\right] \quad \text{(S8)}$$

Supplementary Figures

Supplementary Figure S1 | Various patterns wet etched ~200 nm deep into a microscope slide. (**a**) "*kT*", (**b**) "JHU", (**c**) 12 µm circles arranged in a square lattice, (**d**) 20 µm circles arranged in a square lattice, (**e**) 20 µm wide bars, and (**f**) 20 µm circles arranged in a hexagonal close-packed lattice. All scale bars = 50 µm.

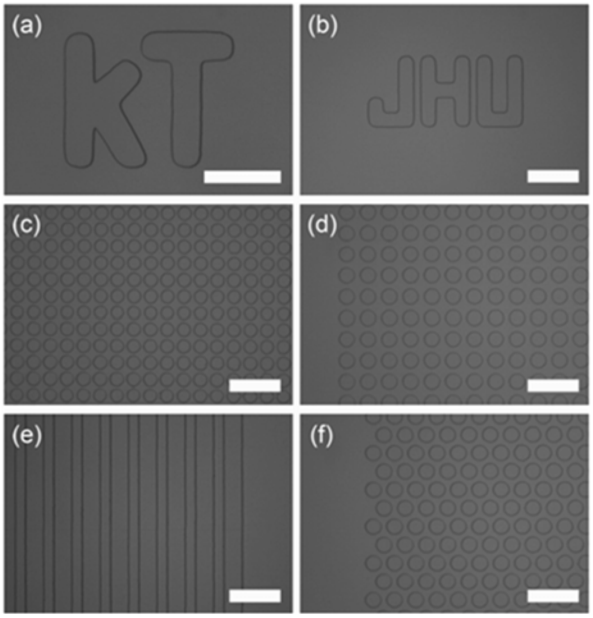

Supplementary Figure S2 | SEM images of ~200 nm deep cross-sectioned etched wells, showing the resulting sidewall geometry. (**a**) Cross-section with an overlaid representation of a 2.2 µm particle; scale bar = 1 µm. (**b**) Magnified cross-section showing the sidewall angle of ~13 °, which was consistent for all etched wells. Both scale bars = 0.5 µm.

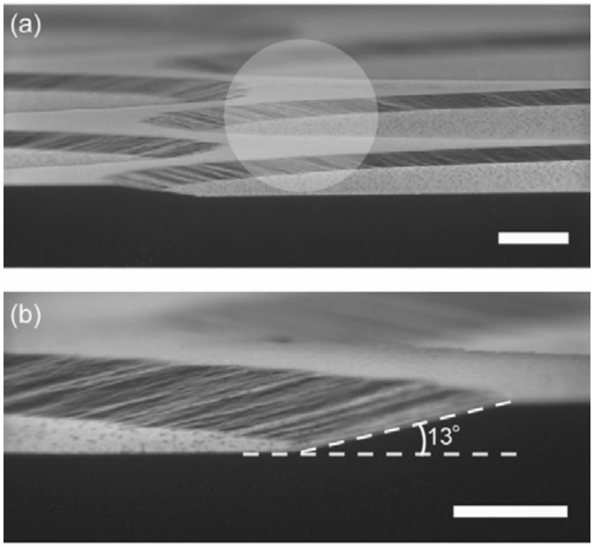

Supplementary Video Captions

Supplementary Video S1 | Excluded volume mediated assembly of NCP ~2 µm diameter SiO_2 colloids on a topographically patterned glass microscope slide at *T* ≅ 25 °C. Circular wells *H* = 285 nm deep arranged in a square lattice combined with ~113 nm diameter PNIPAM hydrogel-mediated depletion attraction cause SiO_2 colloids to assemble along the inside edges of the circular wells. Video was recorded for 2,000 frames and has been sped up 7.2 ×.

Supplementary Video S2 | Same as Supplementary Video S1 except *H* = 90 nm.

Supplementary Video S3 | Same as Supplementary Video S1 except *H* = 35 nm.

Supplementary Video S4 | Reversible excluded volume mediated assembly of NCP ~2 µm diameter SiO_2 colloids on a topographically patterned glass microscope slide at *T* ≅ 25 °C. Circular wells *H* = 285 nm deep arranged in a square lattice combined with ~113 nm diameter PNIPAM hydrogel mediated depletion attraction cause SiO_2 colloids to assemble along the inside edges of the circular wells. Video was extracted from the last 10,000 frames of 50,156 total (30 min) and has been sped up 36 ×.

Supplementary Video S5 | Same as Supplementary Video S4 except *T* = 35 °C (*2L* ≅ 107 nm).

Supplementary Video S6 | Same as Supplementary Video S4 except *T* = 37 °C (*2L* ≅ 53 nm).

Supplementary References

1 Pelton, R. H. & Chibante, P. Preparation of Aqueous Lattices with N-Isopropylacrylamide. *Colloids and Surfaces* **20**, 247-256 (1986).

2 Bell, G. M., Levine, S. & McCartney, L. N. Approximate methods of determining the double-layer free energy of interaction between two charged colloidal spheres. *Journal of Colloid and Interface Science* **33**, 335-359 (1970).

3 Asakura, S. & Oosawa, F. On Interaction between 2 Bodies Immersed in a Solution of Macromolecules. *J. Chem. Phys.* **22**, 1255-1256 (1954).

4 Russel, W. B., Saville, D. A. & Schowalter, W. R. *Colloidal Dispersions*. (Cambridge University Press, 1989).

5 Vrij, A. Polymers at Interfaces and the Interactions in Colloidal Dispersions. *Pure and Appl. Chem.* **48**, 471-483 (1976).

6 Sharma, A. & Walz, J. Y. Direct measurement of the depletion interaction in a charged colloidal dispersion. *Journal of the Chemical Society-Faraday Transactions* **92**, 4997-5004 (1996).

7 Yang, Y., Edwards, T. D. & Bevan, M. A. Modeling depletion mediated colloidal assembly on topographical patterns. *Journal of Colloid and Interface Science* (2014).

8 Bahukudumbi, P. & Bevan, M. A. Imaging Energy Landscapes using Concentrated Diffusing Colloidal Probes. *J. Chem. Phys.* **126**, 244702 (2007).

9 Fernandes, G. E., Beltran-Villegas, D. J. & Bevan, M. A. Spatially Controlled Reversible Colloidal Self-Assembly. *J. Chem. Phys.* **131**, 134705 (2009).
